# Efficient and Precise Grinding of Sapphire Glass Based on Dry Electrical Discharge Dressed Coarse Diamond Grinding Wheel

**DOI:** 10.3390/mi10090625

**Published:** 2019-09-19

**Authors:** Yanjun Lu, Wang Luo, Xiaoyu Wu, Chaolan Zhou, Bin Xu, Hang Zhao, Liejun Li

**Affiliations:** 1 Guangdong Provincial Key Laboratory of Micro/Nano Optomechatronics Engineering, College of Mechatronics and Control Engineering, Shenzhen University, Shenzhen 518060, China; luyanjun@szu.edu.cn (Y.L.); 1810293039@email.szu.edu.cn (W.L.); wuxy@szu.edu.cn (X.W.); zhouchaolan_scut@163.com (C.Z.); 2 School of Mechanical and Automotive Engineering, South China University of Technology, Guangzhou 510640, China; liliejun@scut.edu.cn

**Keywords:** grinding, sapphire glass, coarse diamond grinding wheel, grinding force, surface roughness

## Abstract

In this paper, in view of low grinding efficiency and poor ground surface quality of sapphire glass, the coarse diamond grinding wheel dressed by dry impulse electrical discharge was proposed to perform efficient and precise grinding machining of sapphire glass. The dry electrical discharge dressing technology was employed to obtain high grain protrusion and sharp micro-grain cutting edges. The influences of grinding process parameters such as wheel speed, depth of cut and feed speed on the ground surface quality, grinding force and grinding force ratio on sapphire glass were investigated, and the relationship between grinding force and ground surface quality was also revealed. The experimental results show that the grain protrusion height on the surface of a coarse diamond grinding wheel dressed by dry electrical discharge can reach 168.5 µm. The minimum line roughness *R*_a_ and surface roughness *S*_a_ of ground sapphire glass surface were 0.194 µm and 0.736 µm, respectively. In order to achieve highly efficient ground quality of sapphire glass, the depth of cut was controlled within 7 µm, and the wheel speed and feed speed were 3000–5000 r/min and 10–20 mm/min, respectively. The influences of feed speed and wheel speed on grinding force ratio were more significant, but the influence of depth of cut was little.

## 1. Introduction

Sapphire glass has excellent physical, chemical and mechanical properties such as high temperature resistance, wear resistance, corrosion resistance, high hardness, good light transmission and stable properties. Therefore, it has been widely used in optical electronics, aerospace, display lighting and other fields [1,2,3]. Sapphire glass is a typical hard and brittle material. Although the smooth surface can be obtained by grinding and polishing techniques [4,5,6], it is very difficult to ensure the overall size and shape accuracy of the workpiece due to long polishing time.

Grinding with fine-grained diamond wheels can obtain high surface quality and shape accuracy [7,8,9]. For example, the ground surface qualities of sapphire glass were compared using ceramic-bonded diamond grinding wheels with different diamond grain sizes of 35 μm and 64 μm under the same grinding pressure of 20.5 kPa and rotational speed of ±120 rpm [7]. The elliptical ultrasonic assisted grinding based on #800 resin-bonded diamond grinding wheel was developed to grind the sapphire glass to obtain better ground surface quality [8]. The experimental results showed the stable grinding process area could be extended by about 20% compared with traditional grinding. The #500 electroplated diamond grinding rod was employed to perform micro-groove grinding experiments on sapphire glass surface to investigate the micro-crack formation mechanisms of different crystal planes [9].

However, the fine-grained diamond grinding wheel needed to be frequently dressed and trued [10,11] due to its fast wear, leading to low grinding efficiency. The passivated grains would cause more defects on the ground surface, and the rapid wear of the grinding wheel also easily affected the integral shape accuracy of workpiece [12]. Therefore, the metal-bonded coarse diamond grinding wheel with good wear resistance was proposed to realize high efficiency and precision grinding of sapphire glass due to its large grain protrusion height and sharp micro-grain cutting edges in this paper.

It is very difficult to dress and true the coarse diamond grinding wheel using the traditional mechanical dressing and truing method [13]. Besides, the dressing and truing efficiency was extremely low. Although the laser dressing method could make the diamond grains quickly protrude from the metal bond surface of the grinding wheel [14], the high energy beam would also ablate the diamond grains, which was not conducive to grinding. The previous studies showed that highly efficient dressing of fine-grained diamond grinding wheel can be achieved by using impulse electrical discharge and on-line electrolytic dressing methods [15,16]. However, there has been little research on discharge dressing of a coarse diamond grinding wheel. Therefore, in this paper, the impulse discharge dressing method with simple process and environmental protection was developed to quickly dress the coarse diamond grains of the grinding wheel surface to realize efficient and precise grinding of sapphire glass.

In this paper, the sapphire glass was ground by the coarse diamond grinding wheel dressed using dry electrical discharge technique. The relationship between the grinding force and ground surface roughness of sapphire glass was revealed. The effects of grinding process parameters including wheel speed, depth of cut and feed speed on ground surface quality, grinding force and grinding force ratio of sapphire glass were investigated to optimize the grinding process conditions.

## 2. Experiments and Methods

### 2.1. Dry Electrical Discharge Dressing of the Coarse Diamond Grinding Wheel

**Figure 1:**
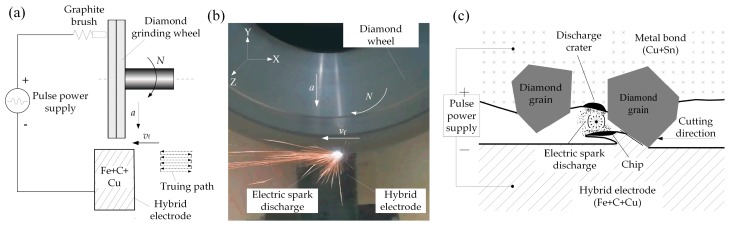
Setup and principle of dry electrical discharge dressing of a diamond grinding wheel: (**a**) schematic diagram; (**b**) dressing photo and (**c**) dressing principle.

The purpose of the dressing of the diamond grinding wheel is to make the diamond grain from wheel surface protrude outside of the bond to form a new sharp micro-grain cutting edge. The pre-developed dry electro-contact discharge dressing and truing technology [17], the high-power ELID (Electrolytic In-Process Dressing) mirror-grinding high-frequency pulse power supply (HDMD, Harbin, China) developed by Harbin Institute of Technology and the truing electrode mixed with cast iron and copper powder (Fe + C + Cu) were employed to dress the #46 coarse diamond wheel with the diameter of 150 mm and thickness of 2.5 mm to obtain a large grain protrusion height on the surface of the diamond wheel. The discharge dressing experiments of the diamond grinding wheel were carried out on a precision CNC (Computer Numerical Control) three-axis grinding machine (SMART-B818III, CHEVALIER, Taiwan). The schematic diagram of electrical discharge dressing is shown in Figure 1a and the photo of discharge dressing is shown in Figure 1b. The positive pole of pulse power supply was connected to the diamond grinding wheel through a graphite brush and the negative pole was connected to the mixed electrode. After grinding wheel cut the mixed electrode, the electric spark discharge was produced between generated chips and metal bond of the wheel surface. Numerous discharge craters were formed on the surface of metal bond to realize the dressing of the wheel. The principle of discharge dressing is shown in Figure 1c. The open-circuit voltage of impulse discharge was 60–120 V and the current was 6.7–20.1 A. After the diamond grinding wheel was dressed, many diamond grains were protruded outside of the metal bond surface to form sharp micro-grain cutting edges. Due to large grain size, it was not easy for the diamond grains to become blunt and fall off. Moreover, the coarse diamond grinding wheel did not need frequent dressing and truing, which can ensure the continuous processing for a long time. Therefore, it had high grinding efficiency and a long useful lifespan, and therefore was more economical in comparison to a fine-grained diamond grinding wheel. After the wheel surface was dressed, large grain protrusion height could be produced, which was beneficial to improve the ground surface quality. Therefore, the coarse diamond grinding wheel can be used to perform efficient grinding of workpieces.

### 2.2. Axial Grinding Experiments of Sapphire Glass

**Figure 2:**
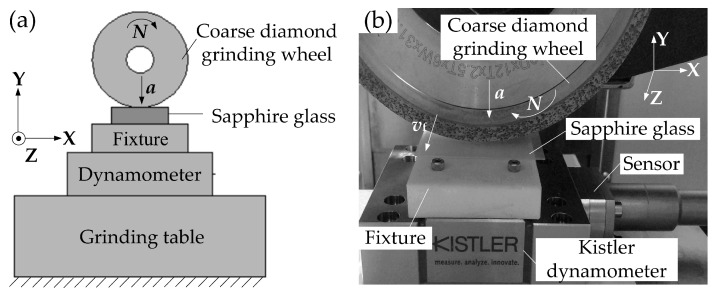
Schematic diagram and photo of axial grinding of sapphire glass: (**a**) schematic diagram and (**b**) grinding photo.

After the diamond grinding wheel was dressed, the axial-feed grinding experiments of sapphire glass were carried out on the three-axis grinding machine (SMART-B818III, CHEVALIER, Taiwan). In the grinding experiments, the synthetic sapphire glass was chosen as a workpiece and its composition was mainly alumina (Al_2_O_3_). The sapphire glass was first bonded to the polymethyl methacrylate (PMMA) plate, and then the plate was fixed on the dynamometer through a fixture. The dressed coarse diamond grinding wheel carried out axial forward reciprocating motion on the surface of sapphire glass. The schematic diagram of axis-grinding is shown in Figure 2a and the grinding photo is shown in Figure 2b. The workpiece size was 30 mm × 15 mm × 0.5 mm. The physical properties are shown in Table 1. The grinding area was 20 mm × 6 mm.

In order to study the influences of different grinding process parameters on the surface grinding force of sapphire glass, the three-way grinding force of sapphire glass was tested during the grinding process, namely, the normal grinding force *F_Y_* perpendicular to the grinding surface and workpiece, the tangential grinding force *F_X_* along the tangential direction of wheel speed and the axial grinding force *F_Z_* parallel to the feed direction of the workpiece (see Figure 2a). The schematic diagram and photo of axial-feed grinding of sapphire glass are shown in Figure 2. The Kistler dynamometer and sensor were used to store the grinding force signal to the computer through the TNS-DES07 data acquisition system. The acquisition time period was 60–90 s, which included the whole grinding process. The average value of the grinding force signal during the grinding process was regarded as the grinding force value *F* under each grinding process condition.

In order to study the effects of grinding process parameters such as depth of cut *a*, feed speed *v*_f_ and wheel speed *N* on ground surface quality, three different grinding process parameters were chosen, and four variables were set under each process parameter in this paper. Based on the previous experimental results, when the depth of cut *a* = 1 μm, feed speed *v*_f_ = 10 mm/min and wheel speed *N* = 3000 r/min, ground surface quality of sapphire glass was the highest. Therefore, a total of 10 groups of different grinding process conditions were designed experimentally, and the cumulative depth of cut under each set of grinding parameter was 30 μm. The experimental parameter lists are shown in Table 2.

Due to the small depth of cut and the amount of feed, a dynamometer with high measurement accuracy and good dynamic acquisition performance was required. After the analog signal of the actual grinding force was collected during the grinding process, it was converted into an electrical signal by a dynamic resistance strain instrument. The signal was amplified by the charge amplifier and was converted into a three-channel three-way grinding force digital signal collected by the data acquisition card. The digital signal was recorded and stored by the computer software and the three-way grinding force was displayed on the computer terminal.

### 2.3. Measurement

As in our previous experiences [10], the roughness measurement results using the non-contact optical 3D laser scanning microscope were basically the same with those measured by the mechanical hand-held contact roughness meter. After the axial grinding and force measurement experiments of the sapphire glass were completed, the 3D laser scanning microscope (VK-250, Keyence, Osaka, Japan) was used to test the 3D topographies of ground sapphire glass surface and dressed grinding wheel surface to obtain the surface roughness *S*_a_ and line roughness *R*_a_ of the ground surface and grain protrusion height of the wheel surface. The roughness of ground sapphire glass was measured along the axial grinding direction three times for each set of grinding process parameters and the average values of the measured data were regarded as the roughness values *S*_a_ and *R*_a_. The high-resolution scanning electron microscope (SEM, Apreo S, FEI Company, Hillsboro, OR, USA) was employed to detect the microscopic morphologies of diamond grain protrusion of the wheel surface and ground sapphire glass surface. The dynamometer (Kistler, 9119AA1, Winterthur, Switzerland) was used to test the grinding force in the axial grinding of sapphire glass using the dressed coarse diamond grinding wheel. The Kistler dynamometer with high resolution and accuracy was used and its measuring accuracy reached 0.01 N. This fixed horizontal preload dynamometer had a compact design and the measuring range along three directions could be up to 30 kN.

## 3. Results and Discussion

### 3.1. Grain Protrusion of the Coarse Diamond Grinding Wheel Surface

**Figure 3:**
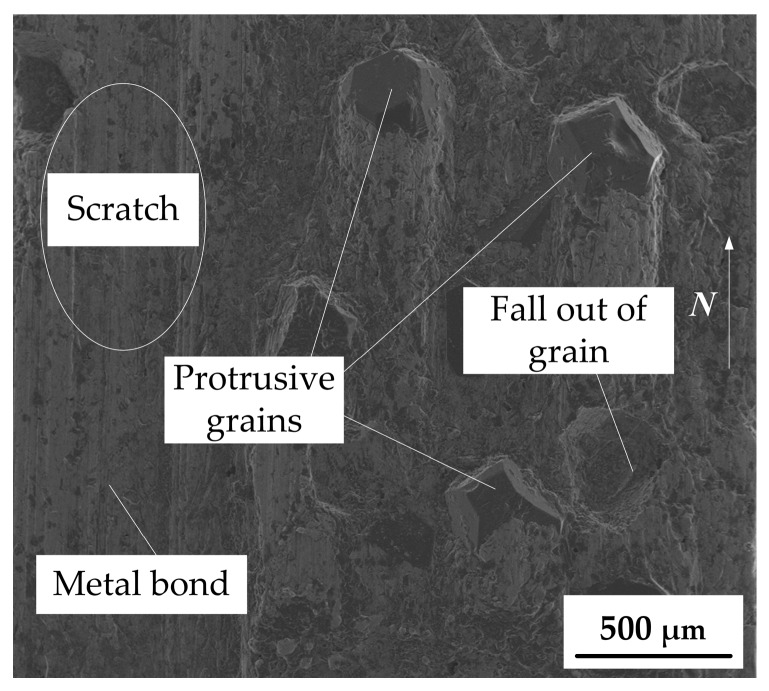
Scanning electron microscope (SEM) photo of dressed coarse diamond grinding wheel surface.

The grain protrusion characteristics of the diamond wheel surface directly affect the grinding quality and processing efficiency of the sapphire glass surface. After the grinding wheel was dressed by dry electrical discharge, the grain protrusion topography of the grinding wheel surface is shown in Figure 3. It can be seen that most of the diamond grains have protruded from the metal bond surface to form sharp micro-grain cutting edges. Meanwhile, it can be seen that there was a discharge crater due to the diamond grain falling out, which was mainly caused by excessive electrical discharge dressing, indicating that the electric spark discharge occurred on the surface of the grinding wheel. Besides, some scratches were found on the surface of the grinding wheel. The possible reason was that there was an extrusion process between the diamond grinding wheel surface and the abrasive grains derived from the mixed electrode. It also further verified the principle of electrical contact discharge dressing shown in Figure 1c.

**Figure 4:**
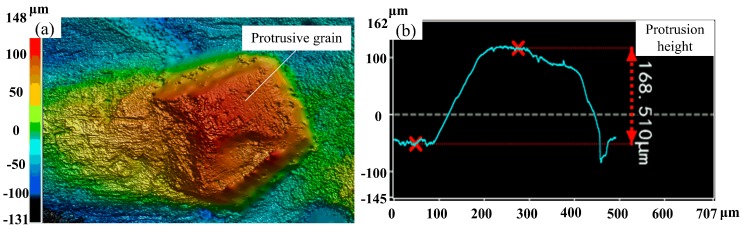
3D topography and protrusion height detection of diamond grain: (**a**) 3D topography of diamond grain protrusion and (**b**) protrusion height detection.

As seen from Figure 3, the distribution of diamond grains was random. Although some grains had fallen out, the overall cutting performance of the grinding wheel was not affected in macroscopic view. After the diamond wheel was dressed, the grain protrusion height of the grinding wheel surface needed to be detected. Figure 4 shows the 3D topography and protrusion height profile of a single diamond grain after the coarse diamond grinding wheel was dressed. Figure 4a shows the 3D topography of the single diamond grain protrusion. It is shown that the protrusion height of diamond grain was about 168.5 μm through measured profile curve of grain protrusion (see Figure 4b). Since the average size of #46 diamond grain was about 350 μm, the grain protrusion height can reach 48% of the theoretical grain size after electrical discharge dressing. According to the literature [20], after the traditional mechanical dressing, the grain protrusion height was only about 1/3 of the theoretical grain size. Therefore, compared with the traditional mechanical dressing, the dry electrical discharge dressing technique not only had higher dressing efficiency, but also could achieve a higher grain protrusion, which was conducive to continuous and efficient grinding. Large grain protrusion height can produce large chip space, which can reduce the accumulation of chips in the grinding area, leading to reductions in friction and grinding force and an improvement in ground surface quality.

### 3.2. Surface Roughness of Ground Sapphire Glass

**Figure 5:**
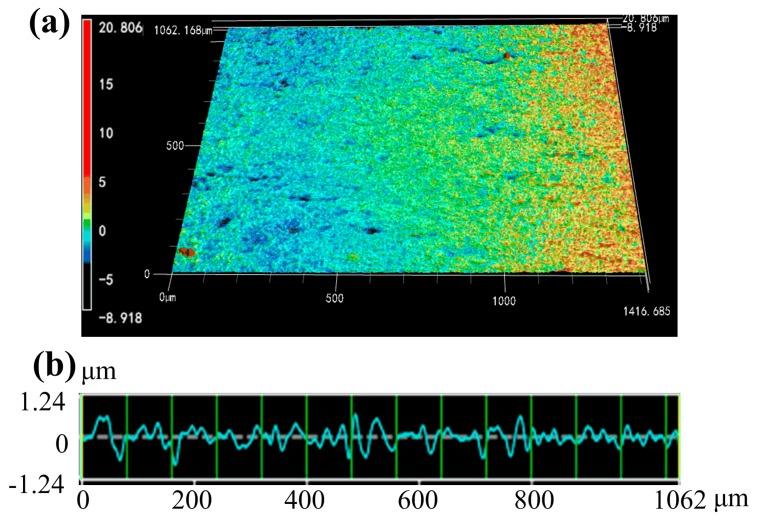
3D topography and roughness curve of ground sapphire glass surface: (**a**) 3D topography and (**b**) roughness curve.

The non-contact optical 3D laser scanning microscope was used to measure the surface roughness of the ground sapphire glass surface. The roughness profile curve can be extracted from the 3D topography using the analysis software. The surface roughness *S*_a_ and line roughness *R*_a_ of the ground surface can be obtained through the measured 3D topography and profile curve of sapphire glass. Figure 5 shows the 3D topography and roughness curve of ground sapphire glass surface. When the grinding process parameters were depth of cut *a* = 1 μm, feed speed *v*_f_ = 15 mm/min and wheel speed *N* = 3000 r/min, the surface roughness *S*_a_ and line roughness *R*_a_ of ground sapphire glass were 0.736 μm (Figure 5a) and 0.194 μm (Figure 5b), respectively. It can be deduced that efficient and precise grinding of sapphire glass based on a coarse diamond grinding wheel dressed by dry electrical discharge was feasible.

**Figure 6:**
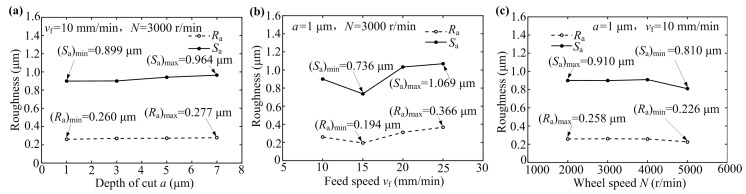
Effects of different grinding process parameters on surface roughness of sapphire glass: (**a**) depth of cut *a*; (**b**) feed speed *v*_f_ and (**c**) wheel speed *N*.

Figure 6 shows the effects of grinding process parameters such as depth of cut *a*, feed speed *v*_f_ and wheel speed *N* on the surface roughness *S*_a_ and line roughness *R*_a_ of the ground sapphire glass surface. It can be seen that the surface roughness *S*_a_ and line roughness *R*_a_ only slightly increased with the increase of depth of cut *a* (see Figure 6a). When the depth of cut *a* = 1 μm, the roughness *S*_a_ and *R*_a_ of ground sapphire glass surface were 0.899 μm and 0.260 μm, respectively. When the depth of cut *a* gradually increased to 7 μm, the roughness *S*_a_ and *R*_a_ of ground sapphire glass surface were 0.964 μm and 0.277 μm, respectively. Although the depth of cut increased by 6 μm, the surface roughness and line roughness only increased by 0.065 μm and 0.017 μm, respectively. This is because the coarse diamond grinding wheel had a large grain protrusion height and chip space and could maintain a sharp grain cutting edge, which did not easily damage the workpiece surface. This reflected excellent grinding performance of the coarse diamond grinding wheel. Therefore, in the precision grinding of sapphire glass using a dressed coarse diamond grinding wheel, the depth of cut may be appropriately increased to effectively improve the grinding efficiency.

As shown in Figure 6b, the roughness of ground sapphire glass surface first decreased substantially and then increased with the increase of feed speed *v*_f_. When the feed speed was 10 mm/min, the number of diamond grains involving grinding per unit time became less so that the grinding force of a single grain became large, resulting in large surface roughness on the sapphire glass surface. If the feed speed was too fast, it would cause inadequate removal of diamond grains for the sapphire glass surface, leading to a collision with workpiece surface and producing many micro-pits and cracks. When the feed speed *v*_f_ was 15 mm/min, the roughness values *S*_a_ and *R*_a_ of the ground surface reached minimum values of 0.736 μm and 0.194 μm, respectively. The *R*_a_ of 0.194 μm was much less than the 0.392 μm value obtained by using fine-grained diamond grinding wheels [5]. Therefore, in the grinding of sapphire glass using a dressed coarse diamond grinding wheel, the suitable feed speed should be set as 15 mm/min.

As seen from Figure 6c, the roughness of ground sapphire glass surface was slightly reduced with the increase of wheel speed *N*. When the wheel speed reached 5000 r/min, the roughness values *S*_a_ and *R*_a_ of ground sapphire glass were 0.810 μm and 0.226 μm, respectively, which were the lowest values recorded. This is because the higher the wheel speed was, the greater the number of diamond grains involved in grinding per unit time were, leading to a reduced depth of cut of a single grain and reduced surface roughness of the ground surface. The results were consistent with the basic grinding principle of hard and brittle materials. Therefore, under the premise of ensuring that the wheel and machine tool did not vibrate, it was necessary to select a wheel speed as high as possible in the grinding process of sapphire glass.

Based on the above experimental results, in the grinding process of sapphire glass using a coarse diamond grinding wheel dressed by dry electrical discharge, it was more advantageous to select a larger depth of cut than a larger feed speed under the premise of ensuring the ground surface quality. The larger depth of cut increased the cutting depth of a single grain to improve the grinding efficiency. Therefore, efficient grinding of sapphire glass may be realized and high ground surface quality may be obtained by choosing a large depth of cut and appropriate feed and wheel speeds.

### 3.3. Surface Topographies of Ground Sapphire Glass 

**Figure 7:**
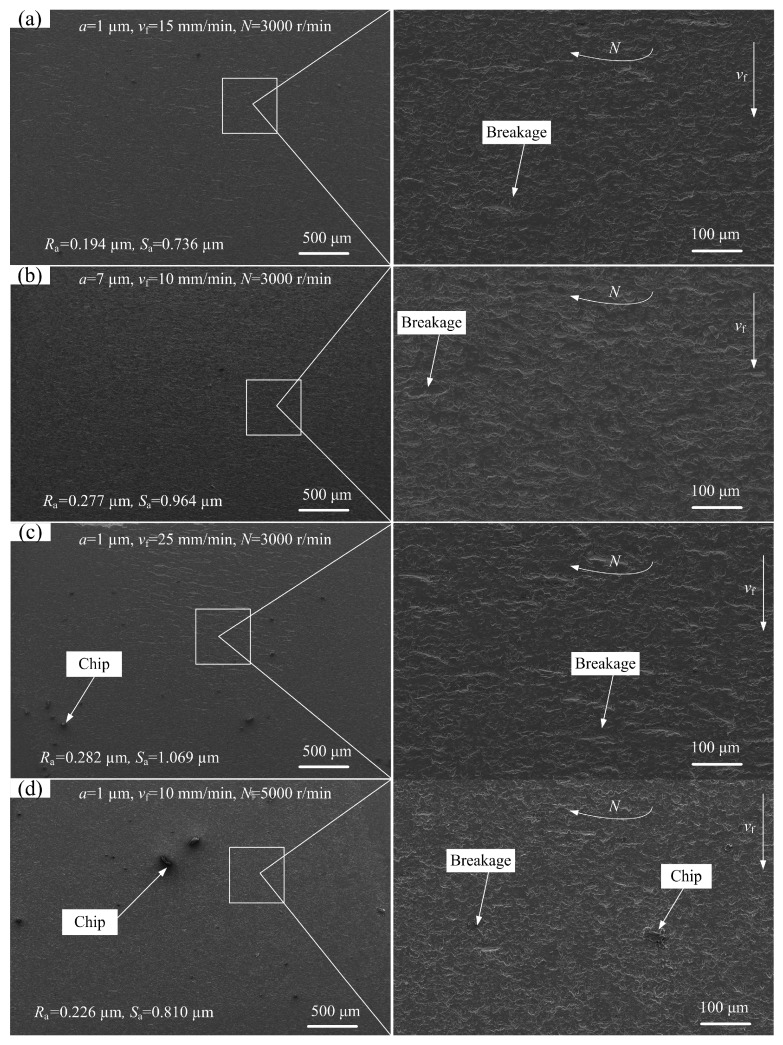
SEM photos of ground sapphire glass surface under different grinding process parameters: (**a**) Experiment No. 5; (**b**) Experiment No. 4; (**c**) Experiment No. 7 and (**d**) Experiment No. 10.

In order to study the effects of different grinding process parameters on surface quality of ground sapphire glass, the microscopic topographies of axial-ground surface were observed and analyzed. Figure 7 shows the SEM photos of ground sapphire glass surface under different grinding process parameters. Comparing Figure 7a,b, when the depth of cut increased and feed speed decreased, the surface roughness *S*_a_ and line roughness *R*_a_ of ground sapphire glass increased, leading to degradation in overall quality of the ground surface. Moreover, too much cutting removal would cause some breakages on the ground surface. Comparing Figure 7a,c, it can be seen that the roughness values *S*_a_ and *R*_a_ of ground sapphire glass greatly increased with the increase of feed speed, indicating that the feed speed had a greater influence on the ground surface quality than the depth of cut. According to the basic grinding principle of hard and brittle materials [21], increasing the wheel speed will increase the number of diamond grains involved in grinding per unit time so that the cutting amount of a single grain will decrease, resulting in reduction of both grinding force and surface roughness. Comparing Figure 7a,d, it is found that when the wheel speed increased and feed speed decreased, the surface quality of ground sapphire glass declined, demonstrating that the feed speed had a greater influence on the ground surface quality than the wheel speed. Therefore, it is necessary to choose appropriate feed speed in precision grinding of sapphire glass.

As a result, moderate feed speed and wheel speed may maintain the ground surface quality, and the depth of cut can be increased to achieve efficient grinding of sapphire glass. Therefore, selecting proper grinding process parameters can improve the material removal rate.

### 3.4. Grinding Force of Sapphire Glass Surface

**Figure 8:**
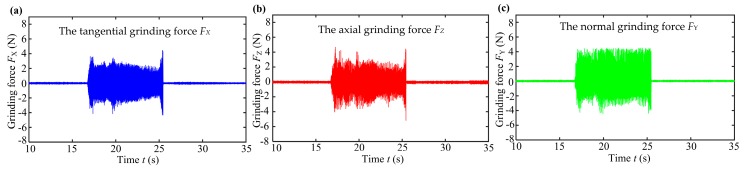
Testing curve of grinding force *F* on sapphire glass surface: (**a**) tangential grinding force *F_X_*; (**b**) axial grinding force *F_Z_* and (**c**) normal grinding force *F_Y_*.

**Figure 9:**
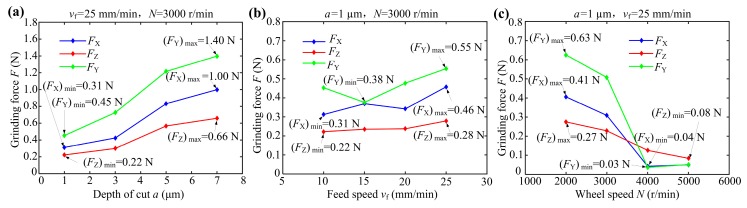
The grinding force *F* of sapphire glass surface versus grinding process parameters: (**a**) depth of cut *a*; (**b**) feed speed *v_f_* and (**c**) wheel speed *N*.

In order to investigate the effects of different grinding process parameters on grinding force of sapphire glass surface, the Kistler dynamometer was used to collect the grinding force signal. The relationship between grinding force and time period can be obtained through filtering. The arithmetic average filtering was used to eliminate the noise points derived from the collected original data before calculating the grinding force. Because the measured grinding force waveform signal had an average value over the period, the signal fluctuated up and down around a certain range of values. In the experiment, the average value of absolute value of peak (valley) of grinding force was regarded as the average grinding force under each set of grinding process parameters. Figure 8 shows the grinding force testing curves along three directions under the grinding condition of depth of cut *a* = 1 μm, feed speed *v*_f_ = 25 mm/min and wheel speed *N* = 3000 r/min. The change curves of the tangential grinding force *F_X_*, axial grinding force *F_Z_* and normal grinding force *F_Y_* versus the time period were also obtained. Thus, the grinding force values under each group of grinding process parameters can be obtained as shown in Figure 9.

Figure 9 shows the effects of different grinding process parameters on the grinding force of sapphire glass surface. As shown in Figure 9a, the normal grinding force *F*_Y_, axial grinding force *F_Z_* and tangential grinding force *F_X_* all increased with the increase of the depth of cut *a*. It can be also seen that under the same grinding process conditions, the normal grinding force was the largest, followed by the tangential grinding force, and the axial grinding force was the smallest. This is mainly because the cutting removal depth of diamond grains played a major role in the grinding force. The cutting removal of the workpiece material was mainly along the tangential direction of the grinding wheel, which conforms to the basic grinding principle of hard and brittle materials. When the depth of cut *a* increased from 1 μm to 7 μm, the normal grinding force *F_Y_*, tangential grinding force *F_X_* and axial grinding force *F_Z_* increased from 0.45 N to 1.4 N, 0.31 N to 1.0 N and 0.22 N to 0.66 N, respectively. Although the depth of cut of diamond grain increased, the grinding force had no obvious change. Hence, the ground surface quality was basically the same. This can be used to explain the results of Figure 6a. It is confirmed that the proper increase of depth of cut had little effects on the grinding force and ground surface quality of sapphire glass, which can effectively improve the grinding efficiency.

As shown in Figure 9b, the normal grinding force *F*_Y_ first decreased and then increased with the increase of feed speed. As the feed speed increased, both the axial grinding force *F_Z_* and tangential grinding force *F_X_* increased. This is because too fast feed speed increased the amount of cutting of a single diamond grain, resulting in an increase in grinding force. However, smaller feed speed tended to cause insufficient cutting removal and chip evacuation of sapphire glass surface, leading to increase in grinding force and surface roughness. Therefore, the suitable feed speed *v*_f_ should be controlled within 10 to 20 mm/min. At this time, the grinding force was substantially less than 0.5 N.

As shown in Figure 9c, the normal grinding force, axial grinding force and tangential grinding force gradually reduced with the increase of wheel speed. The reason may be that the increasing wheel speed increased the number of diamond grains involved in cutting per unit time to reduce the cutting amount of a single grain, which may realize the plastic removal of sapphire glass and improve the ground surface quality of workpiece. However, too fast of a wheel speed would cause vibration of the machine tool system, leading to an increase in grinding error. Therefore, a suitable wheel speed *N* was 3000 to 5000 r/min. This is basically consistent with the roughness results shown in Figure 6.

### 3.5. Grinding Force Ratio of Sapphire Glass Surface

Grinding force reflected the interaction between the grinding wheel and workpiece in the grinding area, which was directly related to the material removal mechanism during the grinding process, ground quality of workpiece and wear of the grinding wheel. The grinding force ratio can directly reflect the cutting condition of the diamond grains cutting into the workpiece surface, namely, the degree of friction between the diamond grains and workpiece surface. By changing the grinding process parameters to control the grinding force ratio to reduce the frictional wear between the diamond grains of the grinding wheel and sapphire glass, its ground surface quality may be improved. The grinding force ratio *λ* may be defined as the ratio of the normal grinding force *F_Y_* to tangential grinding force *F_X_* [22]:

(1) $$\lambda =\frac{F_{Y}}{F_{X}}.$$

**Figure 10:**
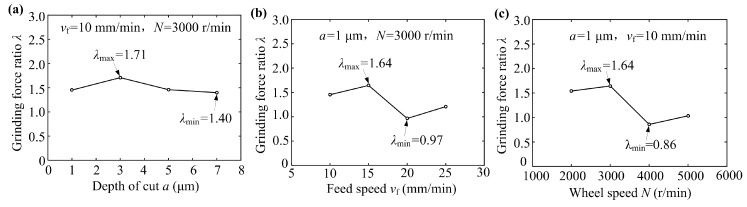
The grinding force ratio *λ* versus grinding process parameters: (**a**) depth of cut *a*; (**b**) feed speed *v*_f_ and (**c**) wheel speed *N*.

Figure 10 shows the relationships between different grinding process parameters and grinding force ratio λ. As seen from Figure 10a, when feed speed *v*_f_ was 10 mm/min and wheel speed *N* was 3000 r/min, the grinding force ratio *λ* ranged from 1.40 to 1.71. The grinding force ratio basically did not change with the increase of depth of cut *a*. It can be seen that the grinding force ratio *λ* varied with the change of feed speed, ranging between 0.97 and 1.64 (see Figure 10b). When the feed speed *v*_f_ was 15 mm/min, the grinding force ratio *λ* reached a maximum value of 1.64. As the feed speed continued to increase, the grinding force ratio decreased so that the cutting amount per unit time and cutting thickness of a single grain increased, leading to an increase in ground surface roughness and grinding force, which were consistent with the results shown in Figure 6b and Figure 9b. As shown in Figure 10c, when the wheel speed increased, the grinding force ratio *λ* also varied from 0.86 to 1.64. Based on the above results, the influences of feed speed and wheel speed on the grinding force ratio were significant, but the depth of cut had little effect.

## 4. Conclusions

In this paper, the #46 metal-bonded coarse diamond grinding wheel dressed by dry electrical discharge is proposed to perform precision axial grinding of sapphire glass. The relationships between ground surface roughness, grinding force and grinding process parameters such as the depth of cut, feed speed and wheel speed are investigated to realize efficient removal of sapphire glass. This paper provides a feasible solution for efficient and precise machining of hard and brittle materials such as sapphire glass. The main conclusions can be summarized as follows:

(1) Using the dry electrical discharge dressing technique, the grain protrusion height of #46 coarse diamond grinding wheel can reach 168.5 μm, which is about 48% of the theoretical diamond grain size. The large grain protrusion height and sharp micro-grain cutting edges can ensure efficient grinding machining for hard and brittle materials.
(2) The minimum roughness *R*_a_ of ground sapphire glass surface is 0.194 μm using the proposed coarse diamond grinding method, which is much less than the obtained *R*_a_ of 0.392 μm using fine-grained diamond grinding wheels [5]. At this point, the normal grinding force reaches a minimum value of 0.38 N and the grinding force ratio achieves a maximum value of 1.64.
(3) The depth of cut is controlled within 7 μm, and the wheel speed and feed speed are maintained between 3000–5000 r/min and 10–20 mm/min respectively, which can realize high efficiency and quality grinding of sapphire glass.
(4) The change of the grinding process parameters will cause the changes of grinding force and grinding force ratio, thus affecting the ground surface quality of the workpiece. In the grinding of sapphire glass, the normal grinding force is the largest, followed by the tangential grinding force, and the axial grinding force is the smallest. The influences of the feed speed and wheel speed on the grinding force ratio are more significant, but the depth of cut has little effect.

## Figures and Tables

**Table 1:** Physical properties of sapphire glass [18,19].

Physical Characteristics	Sapphire Glass
Density, *ρ* (g/cm^3^)	3.98
Mohs’ scale of hardness, *H*	9
Poisson’s ratio, *μ*	0.25–0.3
Shear modulus, *G* (GPa)	145
Elastic modulus, *E* (GPa)	431
Fracture toughness, *K*_IC_ (MPa·m^1/2^)	2.5

**Table 2:** Precision grinding process parameters of sapphire glass.

Term No.	Depth of Cut *a* (μm)	Feed Speed *v*_f_ (mm/min)	Wheel Speed *N* (r/min)
1	1	10	3000
2	3	10	3000
3	5	10	3000
4	7	10	3000
5	1	15	3000
6	1	20	3000
7	1	25	3000
8	1	10	2000
9	1	10	4000
10	1	10	5000
